# Rapid Seedless Synthesis of Gold Nanoplates with Microscaled Edge Length in a High Yield and Their Application in SERS

**DOI:** 10.1007/s40820-016-0092-6

**Published:** 2016-05-11

**Authors:** Sheng Chen, Pengyu Xu, Yue Li, Junfei Xue, Song Han, Weihui Ou, Li Li, Weihai Ni

**Affiliations:** 1 Department of Chemistry, College of Sciences, Shanghai University, Shanghai, 200444 People’s Republic of China; 2 Division of i-Lab & Key Laboratory for Nano-Bio Interface Research, Suzhou Institute of Nano-Tech & Nano-Bionics, Chinese Academy of Sciences, Suzhou, 215123 Jiangsu People’s Republic of China

**Keywords:** Gold nanoplates, Seedless synthesis, SERS, CTAB

## Abstract

We report a facile and reproducible approach toward rapid seedless synthesis of single crystalline gold nanoplates with edge length on the order of microns. The reaction is carried out by reducing gold ions with ascorbic acid in the presence of cetyltrimethylammonium bromide (CTAB). Reaction temperature and molar ratio of CTAB/Au are critical for the formation of gold nanoplates in a high yield, which are, respectively, optimized to be 85 °C and 6. The highest yield that can be achieved is 60 % at the optimized condition. The synthesis to achieve the microscaled gold nanoplates can be finished in less than 1 h under proper reaction conditions. Therefore, the reported synthesis approach is a time- and cost-effective one. The gold nanoplates were further employed as the surface-enhanced Raman scattering substrates and investigated individually. Interestingly, only those adsorbed with gold nanoparticles exhibit pronounced Raman signals of probe molecules, where a maximum enhancement factor of 1.7 × 10^7^ was obtained. The obtained Raman enhancement can be ascribed to the plasmon coupling between the gold nanoplate and the nanoparticle adsorbed onto it.

**Electronic supplementary material:**

The online version of this article (doi:10.1007/s40820-016-0092-6) contains supplementary material, which is available to authorized users.

## Introduction

Noble-metal nanocrystals have attracted a great amount of attention because of their unique light absorbing and scattering properties due to the localized surface plasmon resonance [1–3]. Wet chemical approaches have been developed toward the synthesis of a variety of metal nanocrystals [4, 5], such as nanospheres [6], nanorods [7, 8], nanoplates [9, 10], nanowires [11, 12], etc. Compared to 0D and 1D counterparts, 2D anisotropic nanocrystals, such as gold nanoplates, have large surface areas, sharp corners, and edges which can provide high enhancement of electric field [13–17], and therefore, achieve extensive applications including bio-imaging [18], nanodevices [19], surface-enhanced Raman scattering (SERS) [20], etc.

The growth of gold nanoplates can be directed by either templates or capping agents. By providing constrictions in a 2D space or dimension, planar substrates [14] and interfaces in lamellar bilayer membranes [21] have been used as effective templates for growth of gold nanoplates. Alternatively, capping agents can preferentially adsorb on a specific surface of gold so that the adsorption of gold ions to this surface is blocked, and the growth is restricted on a planar direction. These agents should be surfactants [22], polymers [23–26], biomolecules [27], and halide ions [28, 29]. Among them, cetyltrimethylammonium bromide (CTAB) is one of the most frequently used surfactants for the growth of gold nanoplates, which can be easily adsorbed onto the surface of gold through complexing with halide ions. For example, Mirkin group developed the seed-mediated growth of small gold nanoplates using CTAB as the capping agent [30]. Although the seed-mediated synthesis process effectively prohibits secondary nucleation and easily controls the size and shape of the final product, it involves multistep growth of seeds. To solve this problem, Huang group developed a seedless approach to synthesize gold nanoplates in the presence of CTAB via thermal reduction, where reaction solutions were preheated before they were mixed together to ensure the control of the size distribution [22, 31]. However, long preheating time will result in higher time consumption for the process and prove to be cost ineffective. Recently, high-yield synthesis of gold nanoplates with submicron edge length was reported where iodide ions were used as both the capping and etching agents [28]. However, rapid synthesis of gold nanoplates in microscaled edge length with high yield, simplicity, and low-cost still remains challenging.

Herein, we report a facile and reproducible approach of rapid, seedless synthesis of single crystalline gold nanoplates with edge lengths in micron orders of magnitude. The reaction was carried out by reducing gold ions with ascorbic acid in the presence of CTAB. The reaction temperature and molar ratio of CTAB/Au on the products were examined in detail. The SERS properties of the as-synthesized gold nanoplates were also investigated.

## Experimental

### Chemicals

Hydrogen tetrachloroaurate tetrahydrate (HAuCl_4_·3H_2_O), L-ascorbic acid (AA), cetyltrimethylammonium bromide (CTAB), and 4-Mercaptophenol (Mph) were purchased from Sigma-Aldrich. All chemicals and reagents were used without any further purification. Ultrapure water was obtained from the Milli-Q system (18.2 MΩ cm^−1^).

### Synthesis of Gold Nanoplates

A typical synthesis procedure is as follows: first, 100 µL of 0.1 M HAuCl_4_ was added into 3 mL of 0.02 M CTAB aqueous solution in a plastic tube, and the mixture was left undisturbed for several minutes. Then, 100 µL of 0.1 M AA was added to the mixture, followed by rapid inversion for 10 s. The resultant solution was immediately placed in a water bath of 85 °C and kept undisturbed for about 1 h. The products were washed by centrifugation at 4000 rpm for 10 min and finally dispersed in deionized water.

### Preparation of SERS Substrates

The SERS substrate was prepared as follows: gold nanoplate solution was drop-casted onto a clean silicon substrate. The substrate was rinsed and blown dry by nitrogen gas. Afterward, it was immersed into a solution of gold nanoparticles for several minutes, allowing deposition of gold nanoparticles on the gold nanoplates. After thoroughly rinsing with water for several times, it was immersed into a solution of 0.01 M Mph for 3 h. The substrate was carefully rinsed and blown dry by nitrogen gas before the SERS measurement. A cross-bar was finally marked on the substrate for locating the gold nanoplate and investigating them individually under the optical microscope and SEM.

### Characterizations

The extinction spectra of the gold nanoplates were recorded using Agilent Cary 60 UV–Vis spectrophotometer using a cuvette having 0.5-cm path length. The morphology of the gold nanoplates was characterized by Hitachi S-4800 field emission and FEI Quanta 250 FEG SEMs. Powder X-ray diffraction (XRD) patterns were recorded on a Bruker D8 Advance powder X-ray diffractometer at a scanning rate of 4° min^−1^, using Cu-Kα radiation (*λ* = 1.54056 Å). Raman scattering spectra were measured on a micro-Raman system (HR evolution 260, Horiba). The sample was excited at 633 nm and 4 mW in the Raman measurement. The Raman scattered light was collected using an Olympus objective (100 *X*, N.A. = 0.9, W.D. = 1 mm). Raman spectra were recorded using a grating of 600 lines per mm with an integration time of 15 s.

## Results and Discussion

**Fig. 1:**
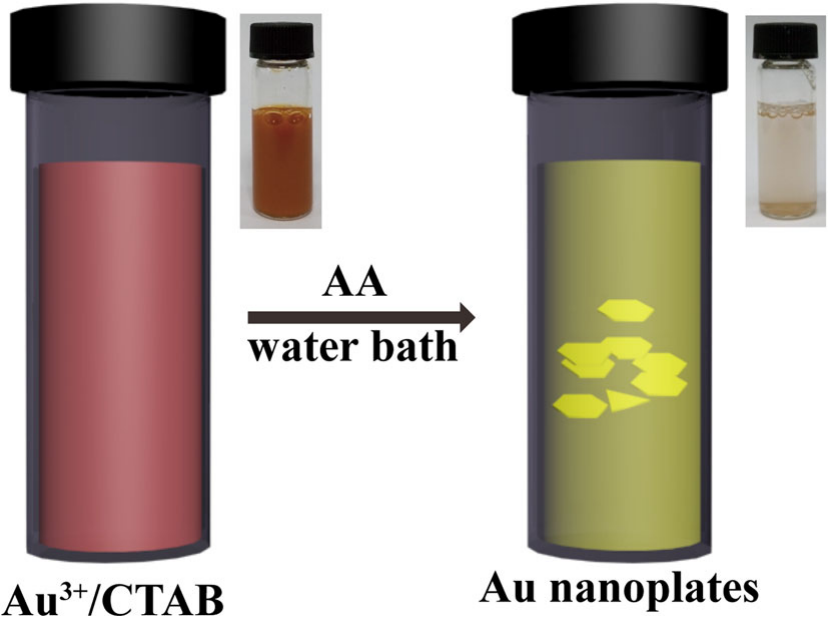
Schematic illustration of the synthesis of the gold nanoplates

As shown in Fig. 1, the synthesis of the gold nanoplates is very straightforward. To be specific, aqueous solutions of HAuCl_4_ and CTAB were first mixed and left undisturbed at room temperature for several minutes. The color of the mixture solution slowly changed to red brown owing to the formation of ${\text{AuBr}}_{4}^{ - }$ complex ions [25, 32, 33]. Afterward, a reducing reagent of AA was injected, which quickly makes the mixture to be colorless. The reaction solution was kept undisturbed in a water bath of 85 °C for 1 h, and some precipitation of the resultant products was found at the bottom of the glass vial.

**Fig. 2:**
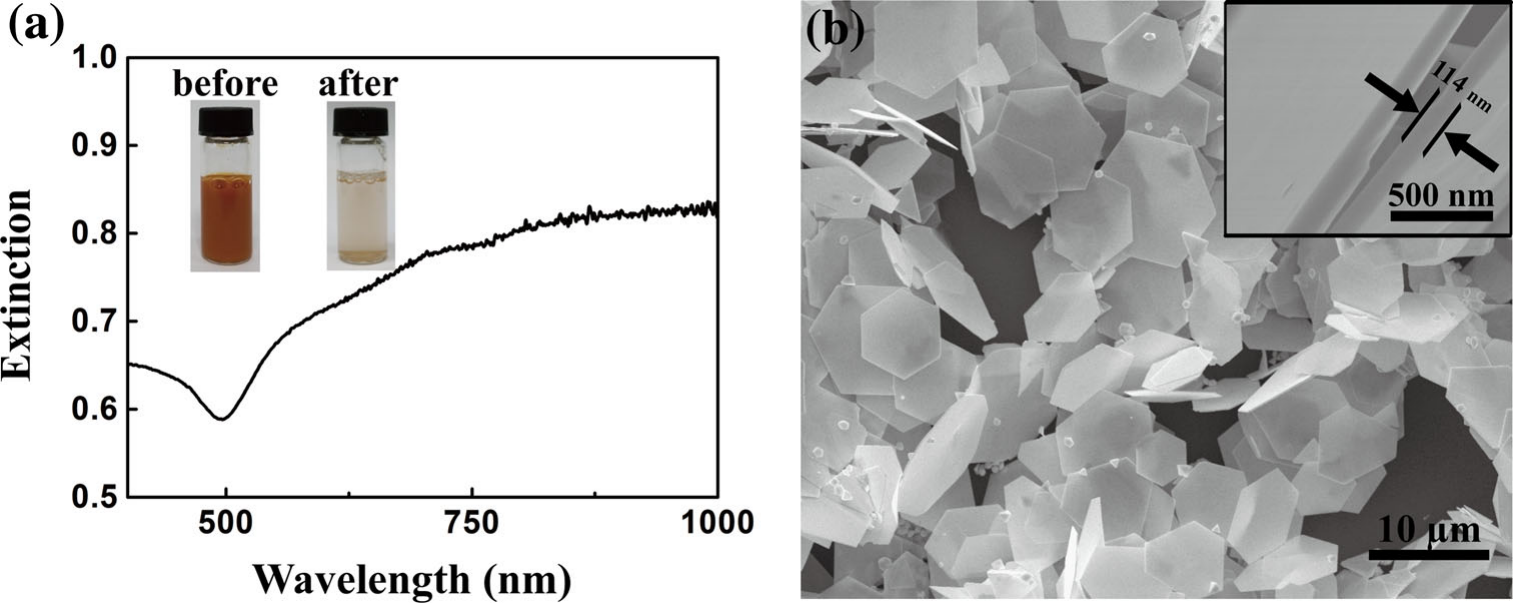
**a** The extinction spectrum of gold nanoplates in aqueous solution. The *inset* shows the photographs of the solutions before and after the growth of gold nanoplates. **b** The SEM images of gold nanoplates. The *inset* shows a cross-sectional SEM image of the gold nanoplates

Figure 2a shows the extinction spectra of the as-prepared gold nanoplates in an aqueous solution, and the inset shows the comparison of the colors of the solution before and after the synthesis reaction. The gold nanoplates exhibit a broad absorption band starting from 500 nm, which can be ascribed to the dipole and quadrupole plasmon resonances [25, 34, 35]. The morphologies of resultant products were examined by SEM (see Fig. 2b) and statistically analyzed, and the results are shown in Fig. S1 of Supporting Information (SI). Although a small amount of spherical nanoparticles were observed as well, the resultant plate-like products were mainly composed of 8.5 % triangular and 91.5 % equilateral hexagonal nanoplates with average edge lengths of 3.5 µm, and thicknesses of around 114 nm (obtained from the tilted SEM image shown in the inset), which strongly confirms the relatively monodisperse and uniform nanoplates.

**Fig. 3:**
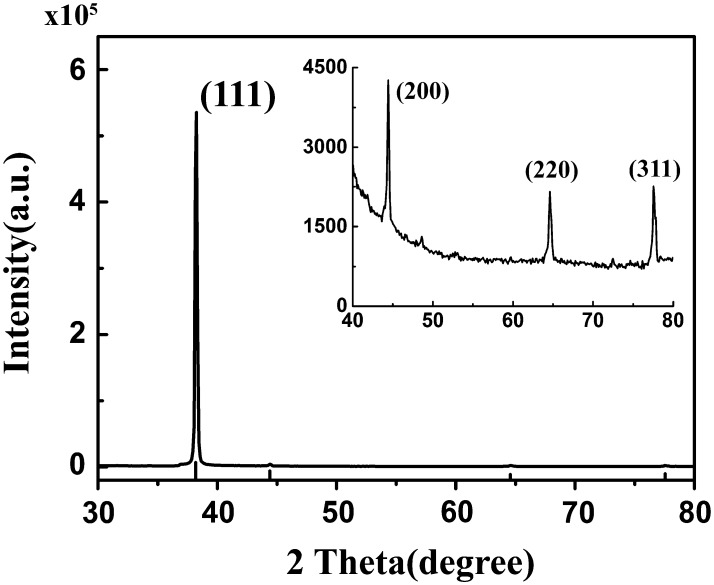
XRD pattern of the as-prepared gold nanoplates deposited on quartz

The XRD pattern of gold nanoplates was recorded using a quartz substrate (Fig. 3). The diffraction peaks are assigned to (111), (200), (220), and (311) planes of face-centered-cubic structure of Au (PDF No. 04-0784). Note that the intensity of Au (111) peak is much stronger than those of (200), (220), and (311). The diffraction intensity ratio of (200)/(111) is extremely lower than the standard file (0.0051 versus 0.52). This XRD result clearly indicates that the as-synthesized gold naonoplates are single crystalline and possess a preferred plane of (111).

**Fig. 4:**
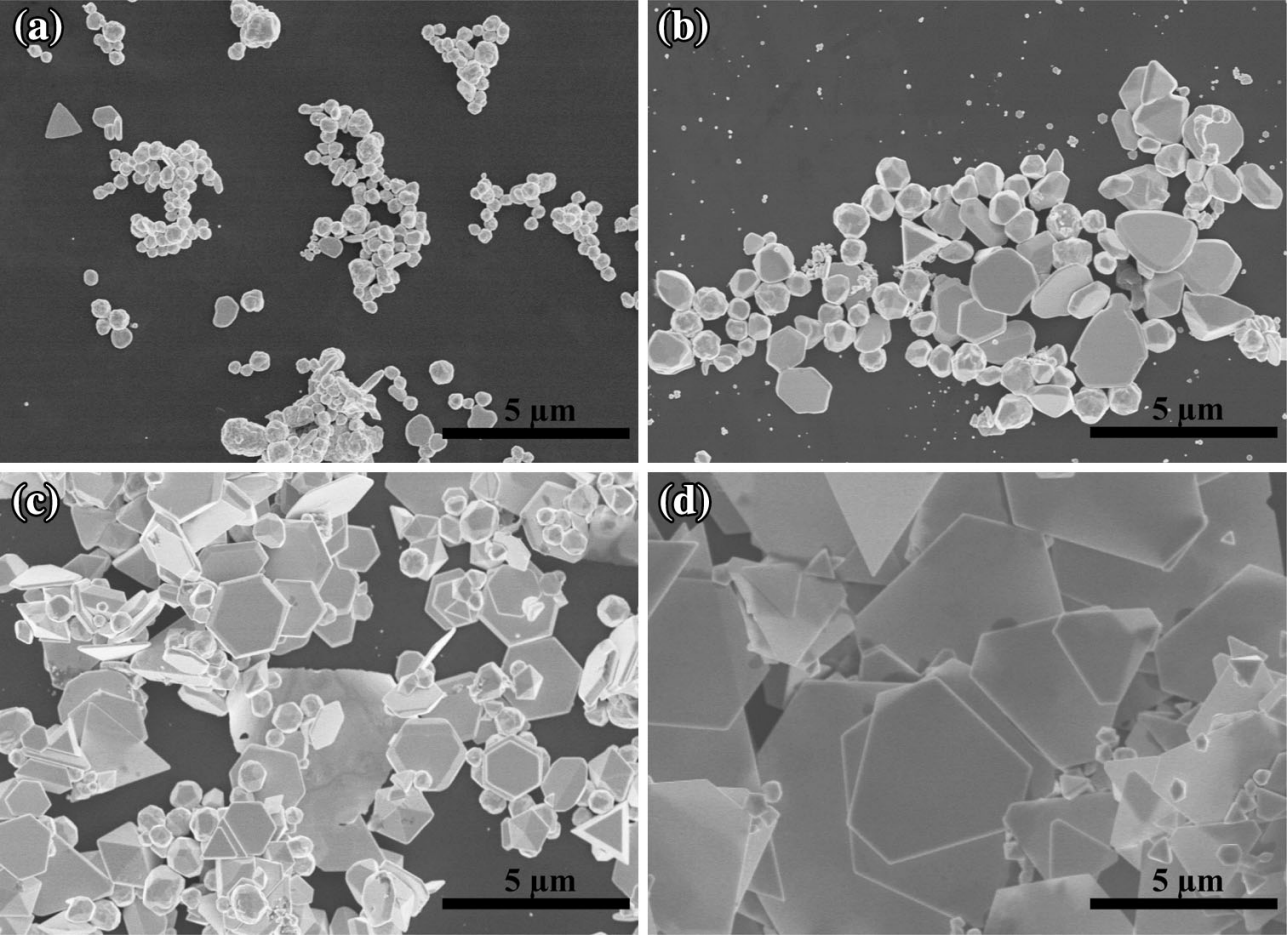
SEM images of the samples prepared at **a** 25 °C, **b** 45 °C, **c** 65 °C, and **d** 95 °C. The molar ratio of Br^−^/Au is fixed at 6:1

To achieve a high yield, the synthesis conditions that affect the growth of gold nanoplates were finely optimized. It is known that the reaction temperature plays an important role in the formation of anisotropic nanostructures [36, 37]. Therefore, in our experiment, the reaction temperature was varied from 25 to 95 °C, as illustrated in Fig. 4. Figure 4a shows the morphologies of resultant products synthesized at room temperature (25 °C) where most of them are identified as plate- and sphere-like particles. Both the yields and the edge lengths of the nanoplates start to increase when the temperature is increased. Figure 4b, c shows the products synthesized at 45 and 65 °C, respectively. At 85 °C, a large amount of hexagonal nanoplates with edge length of about ~3.5 µm can be found where the highest yield of 60 % is obtained (Fig. 2b). The extinction spectrum suggests that increasing the reaction temperature further to 95 °C leads to both inhomogeneous size distribution of the gold nanoplates and the decrease in the yield to 53 % (Fig. 4d). These results indicate that the most preferable reaction temperature for the high-yield synthesis of the gold nanoplates is 85 °C. Extinction spectra of the Au products grown at different temperature are shown in Fig. S1, Supporting Information (SI). In contrast to the turbid appearance of the solutions obtained at 25 and 45 °C, those at 65, 85, and 95 °C exhibit features of plasmon resonances belonging to the gold nanoplates, which is consistent with the SEM images. The gold nanoplate solution obtained at 85 °C shows the highest extinction intensity among the these three, suggesting the highest yield.

**Fig. 5:**
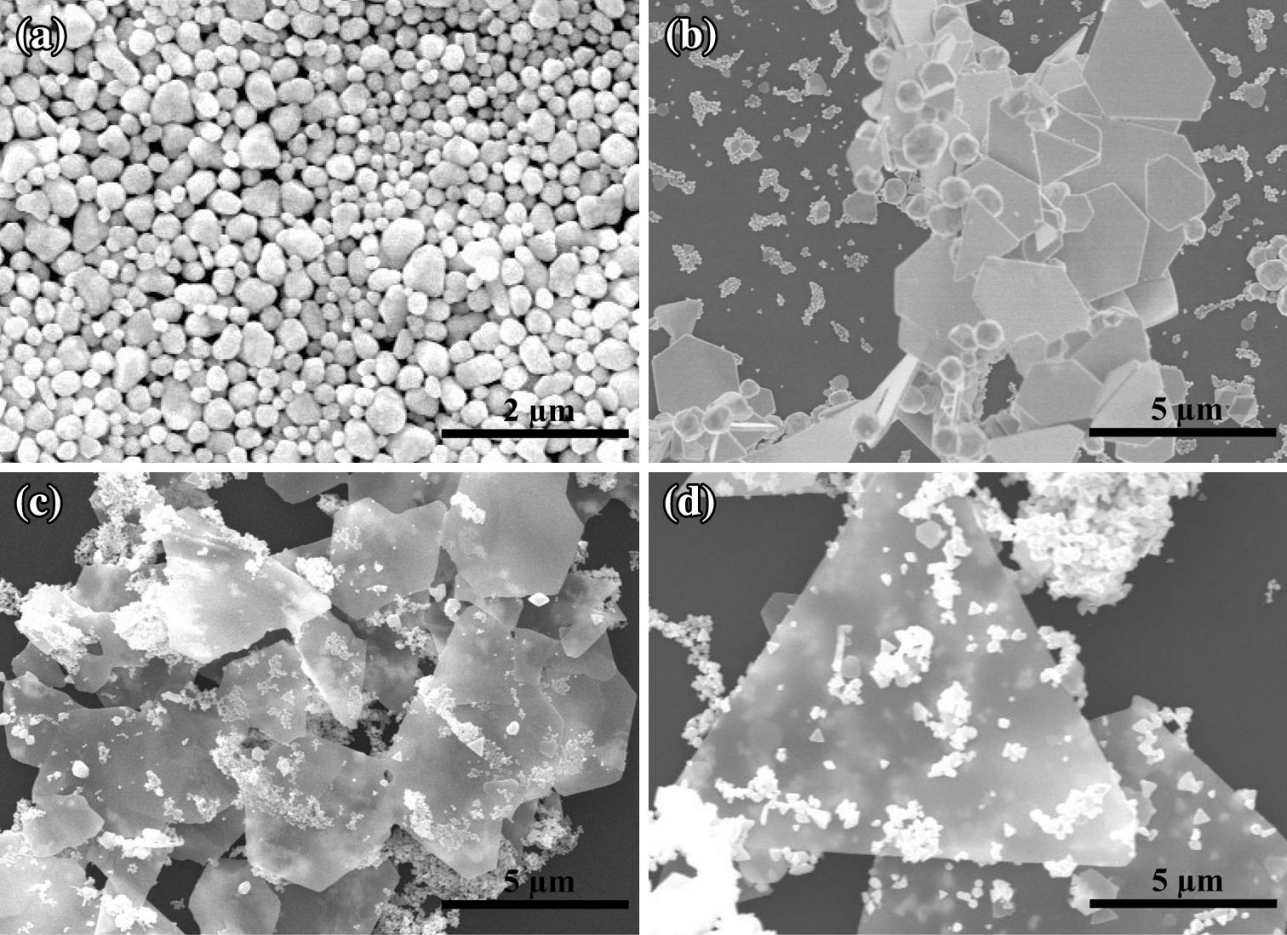
SEM images of the samples prepared with different molar ratios of Br^−^/Au: **a** 1:1, **b** 3:1, **c** 12:1, and **d** 30:1. The reaction temperature is fixed at 85 °C

Besides the reaction temperature, the molar ratio of Br^−^/Au has greatly significant influence on the formation of the gold nanoplates. Figure 5 shows the gold nanoplates synthesized at various Br^−^/Au ratios with the addition of different volumes of 0.2 M NaBr to the reaction solution. The synthesis was carried out in a water bath of 85 °C for 1 h. As shown in Fig. 5a, when molar ratio of Br^−^/Au is 1:1, the final products are almost nanoparticles, and no plate-like nanocrystals were found. Nanoplates start to appear in the resultant products when molar ratio of Br^−^/Au is increased to 3:1 (Fig. 5b). When molar ratio of Br^−^/Au is tuned to 6:1, nanoplates dominate in the resultant products (Fig. 2b). The amount of nanoplates start to decrease when molar ratio of Br^−^/Au is further increased (Fig. 5c, d). These results indicate that the Br^−^/Au molar ratio of 6:1 is the most preferable for the formation of nanoplates. Also, CTA^+^ as the capping agent is necessary in the synthesis, in which a proper concentration of 20 mM was used. The results were also confirmed by the extinction spectra (Fig. S1b). A prominent plasmon peak at around 550 nm can be found for the solutions obtained at Br^−^/Au ratios of 1:1 and 3:1, indicating the presence of considerable amounts of spherical gold nanoparticles in the solutions. This peak becomes less observable at Br^−^/Au ratios of 6:1, and the spectrum in the near infrared increases, suggesting high yield of nanoplates. The extinction intensity drops when the ratio is further increased to 12:1 and 30:1.

**Fig. 6:**
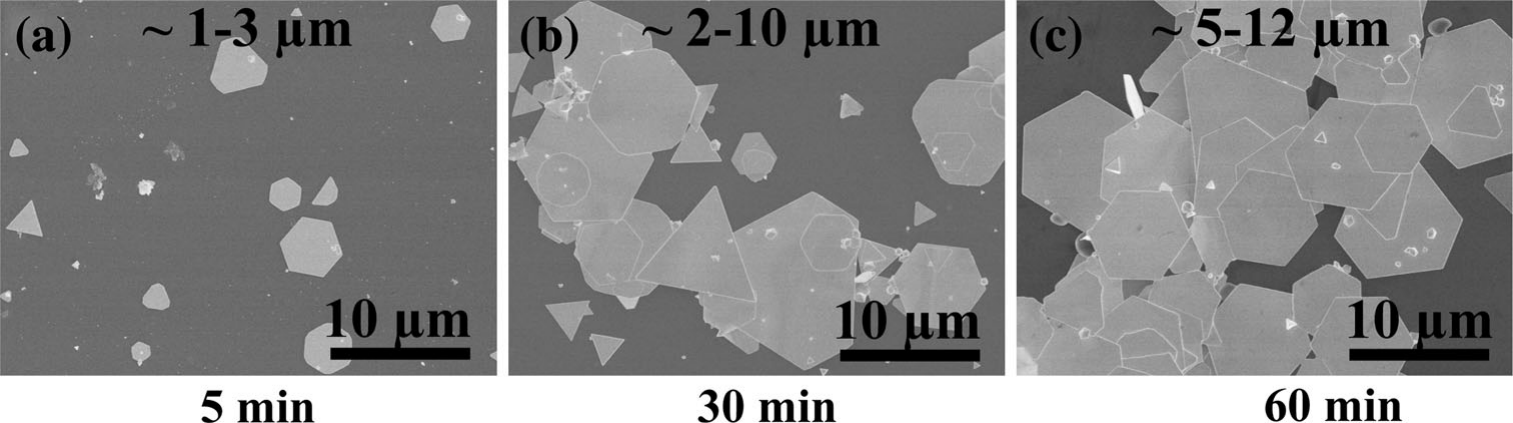
SEM images of the gold nanoplates obtained **a** less than 5 min, **b** within 30 min, and **c** after 60 min

It is generally believed that the formation pathway of nanoplates is “kinetically controlled” along with the surfactant as capping agent or template-like CTAB and PVP [16, 33]. On the basis of the experimental evidence, the reaction temperature of 85 °C and the Br^−^/Au molar ratio of 6:1 are optimal conditions for obtaining high-yield gold nanoplates. To investigate this reaction route, a series of experimental investigations on the formation process were carried out through sampling gold nanoplates with the increasing reaction times. Figure 6 shows the sizes and shapes of gold nanoplates under different increasing reaction times. It can be found that small plate-like nuclei are formed in less than 5 min (Fig. 6a). They grow bigger into nanoplates with micrometer edge length within 30 min (Fig. 6b), and a high yield is obtained after 60 min (Fig. 6c). The experiment was performed at the preferable reaction temperature and concentration of Br^−^ and CTA^+^. This result indicates that under the preferable reaction condition, a minimum 1-h reaction time is essential for the synthesis of gold nanoplates in a high yield.

**Fig. 7:**
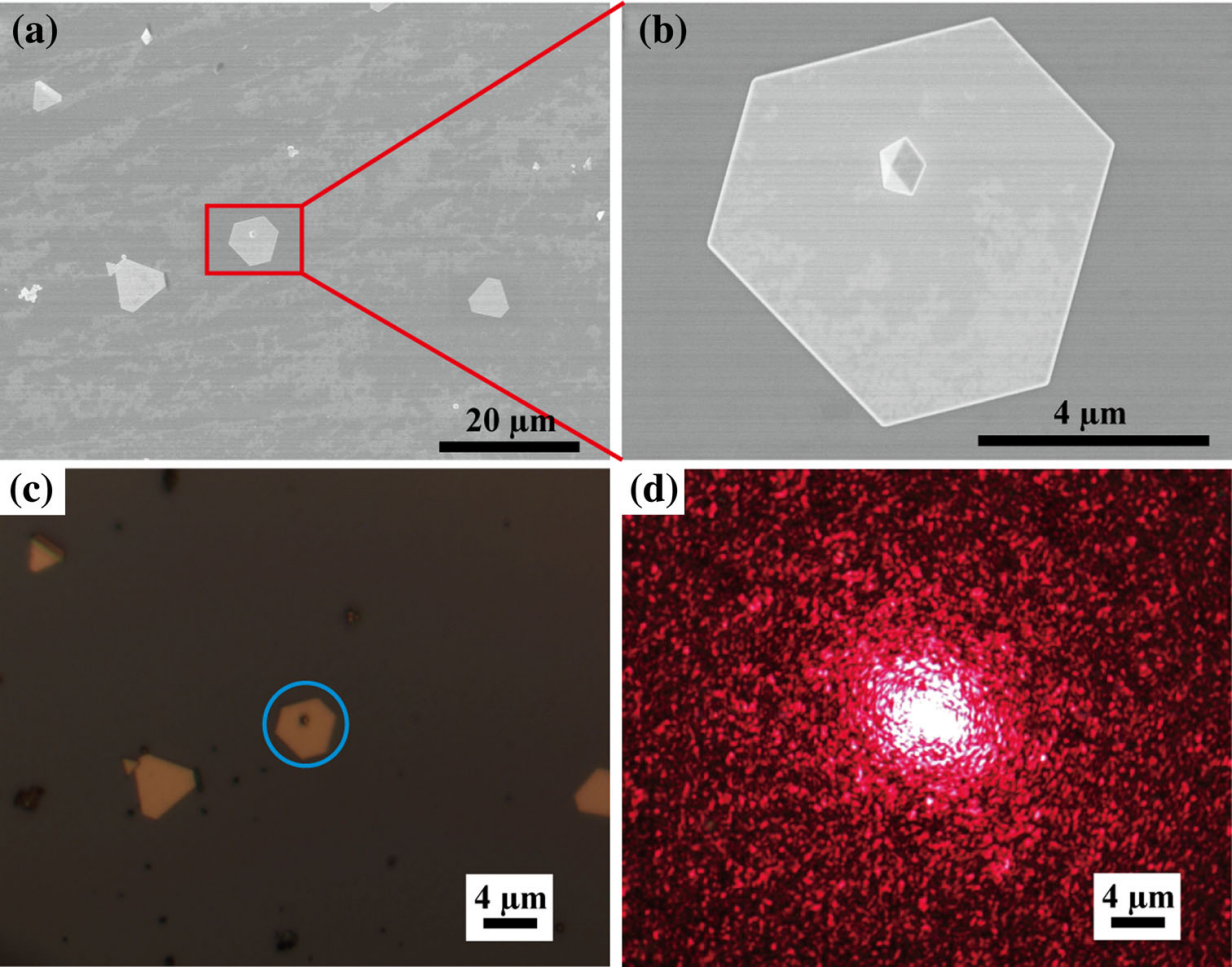
**a** The SEM images of gold nanoplates with a nanoparticle adsorbed. **b** Zoomed-in SEM image of (**a**). **c** Corresponding optical image with the nanoplate highlighted in the *blue circle*. **d** The focal point of the excitation laser positioned on the nanoparticle. (Color figure online)

**Fig. 8:**
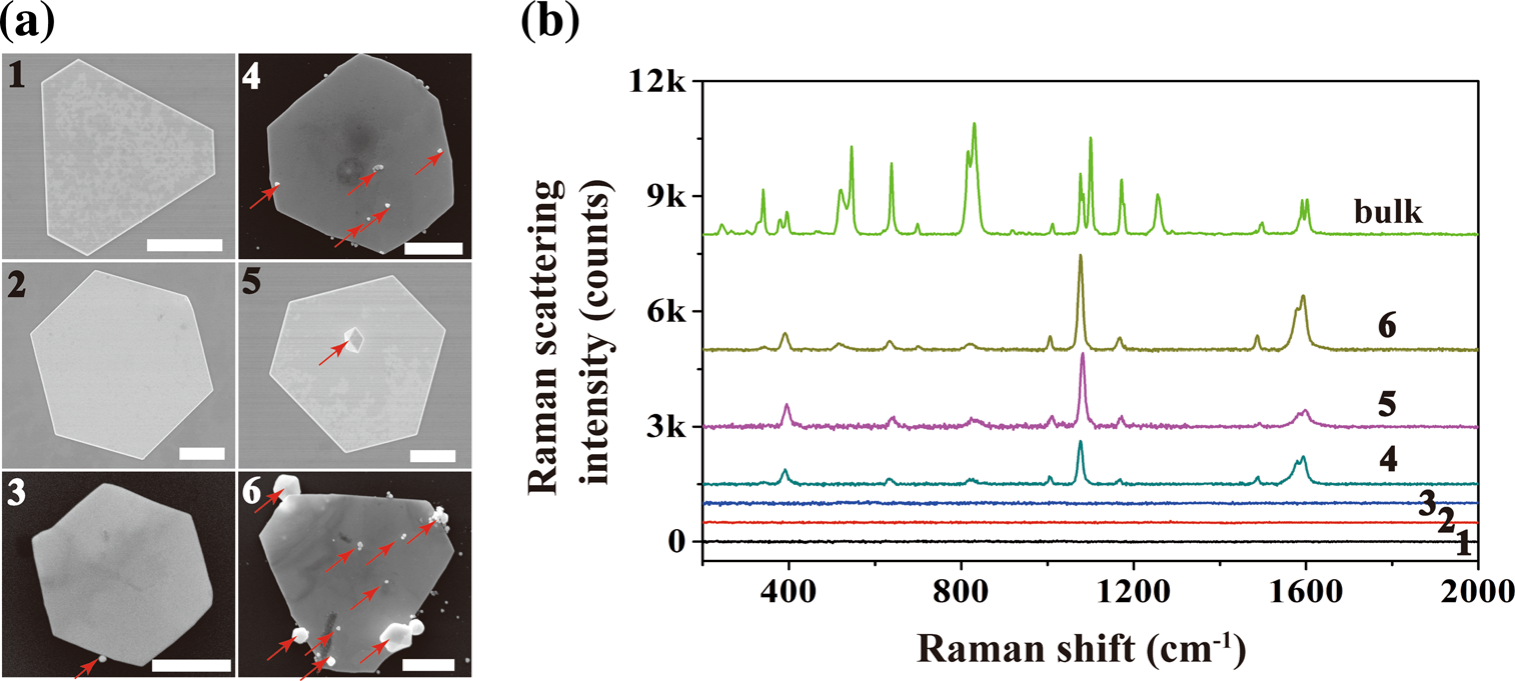
**a** SEM images of gold nanoplates without any adsorbed nanoparticles (cases 1 and 2), those with adsorbed nanoparticles on the side (case 3), and those with the adsorbed nanoparticles on the surface (cases 4, 5, and 6). The nanoparticles are indicated by *red arrows*. The *scale bars* are 2 µm. **b** Raman spectra of bulk MPh and corresponding nanoplates in cases from 1 to 6. (Color figure online)

Recently, many endeavors have been devoted to the fabrication of efficient SERS substrates. For example, the nanoscaled gaps provided by sandwiched structures can greatly augment the Raman signals [38–42]. Individual gold nanoplates, however, are rarely used as SERS substrates alone due to their flat surface. Here, we demonstrate that the gold nanoplates with adsorbed gold nanoparticles can be used as efficient SERS substrates for achieving high enhancement factors (EFs). The Raman measurements were performed under excitation of 633 nm where Mph was used as the probing molecule. The same objective was used for the excitation and collection of the Raman scattering light. Figure 7a, b shows a typical gold nanoplate with adsorbed gold nanoparticle. Under the optical microscope, the same nanoplate was located and positioned in the center of the optical view (Fig. 7c). The focal point of the excitation laser at 633 nm was thereafter positioned exactly on the nanoparticle (Fig. 7d). Raman responses from gold nanoplates with and without adsorbed nanoparticles are measured and compared (Fig. 8a). Raman spectra from the individual corresponding gold nanoplates are shown in Fig. 8b. The Raman signals from nanoplates without nanoparticles adsorbed are so weak that no peaks can be identified on the spectrum (cases 1 and 2). It is also the case when a nanoparticle is adsorbed onto the edge of the nanoplate (case 3). This result is related to the lack of hot spots no matter whatever the shapes of these nanoplates be. Interestingly, strong Raman signals are observable when some nanoparticles are adsorbed on the upper surface of the nanoplates (cases 4, 5, and 6), and the excitation laser beam is focused on the nanoparticles. In such cases, hot spots are formed in the gap between nanoparticles and nanoplates, which greatly enhances the Raman response because of the interparticle electromagnetic coupling [43–45]. Characteristic Raman peaks of Mph can be found on the measured spectra at 830, 1014, 1083, 1175, and 1600 cm^−1^. Among these peaks 830, 1014, and 1083 cm^−1^ are, respectively, assigned to C–H wagging, C–C bending, and C–S stretching, and 1175 and 1600 cm^−1^ are belong to C–H bending modes [43, 46, 47]. Note that the Raman spectra from the SERS substrates are slightly different from the bulk (Fig. 8b).

Enhancement factors (EFs) of the samples are evaluated by the equation of EF = (ISERS/IBulk)(NBulk/NSERS), where ISERS and IBulk represent the Raman intensity values measured on the SERS substrate and bulk Mph, respectively. NSERS and NBulk are the numbers of Mph molecules adsorbed on the SERS and bulk samples inside of the laser spot, respectively. The EFs of SERS peaks at 1011, 1081, 1492, and 1599 cm^−1^ are evaluated to be 1.5 × 107, 4.0 × 106, 1.7 × 107, and 8.1 × 106, respectively. These results suggest that the gold nanoplates can be used as ideal SERS substrates for detecting Raman analytes.

## Conclusions

In summary, we successfully developed a simple but effective route to the synthesis of single crystalline gold nanoplates with edge length on the order of microns. Optimized reaction temperature and molar ratio of CTAB/Au are found to be, respectively, 85 °C and 6:1 for the formation of gold nanoplates in a high yield of 60 %. The synthesis to achieve the microscaled gold nanoplates can be finished in less than 1 h under proper reaction conditions. Therefore, the reported synthesis is time- and cost effective. The gold nanoplates were further employed as the SERS substrates and investigated individually. Interestingly, only those adsorbed with gold nanoparticles exhibit pronounced Raman signals of probe molecules, where a maximum enhancement factor of 1.7 × 10^7^ was obtained. Our work demonstrated that a designed nanostructure consisting of a nanoplate adsorbed with a nanoparticle on its upper surface can be used as an efficient SERS substrate for reproducible enhancement.

## Electronic supplementary material

Below is the link to the electronic supplementary material.

Supplementary material 1 (PDF 334 kb)

## References

[1] El-Sayed MA (2001). Some interesting properties of metals confined in time and nanometer space of different shapes. Acc. Chem. Res..

[2] Zijlstra P, Orrit M (2011). Single metal nanoparticles: optical detection, spectroscopy and applications. Rep. Prog. Phys..

[3] Ni W, Kou X, Yang Z, Wang J (2008). Tailoring longitudinal surface plasmon wavelengths, scattering and absorption cross sections of gold nanorods. ACS Nano.

[4] Koczkur KM, Mourdikoudis S, Polavarapu L, Skrabalak SE (2015). Polyvinylpyrrolidone (PVP) in nanoparticle synthesis. Dalton Trans..

[5] Polavarapu L, Mourdikoudis S, Pastoriza-Santos I, Perez-Juste J (2015). Nanocrystal engineering of noble metals and metal chalcogenides: controlling the morphology, composition and crystallinity. CrystEngComm.

[6] Ruan Q, Shao L, Shu Y, Wang J, Wu H (2014). Growth of monodisperse gold nanospheres with diameters from 20 to 220 nm and their core/satellite nanostructures. Adv. Opt. Mater..

[7] Sau TK, Murphy CJ (2004). Room temperature, high-yield synthesis of multiple shapes of gold nanoparticles in aqueous solution. J. Am. Chem. Soc..

[8] Sau TK, Murphy CJ (2004). Seeded high yield synthesis of short au nanorods in aqueous solution. Langmuir.

[9] Shao Y, Jin Y, Dong S (2004). Synthesis of gold nanoplates by aspartate reduction of gold chloride. Chem. Commun..

[10] Soejima T, Kimizuka N (2009). One-pot room-temperature synthesis of single-crystalline gold nanocorolla in water. J. Am. Chem. Soc..

[11] Huo Z, Tsung CK, Huang W, Zhang X, Yang P (2008). Sub-two nanometer single crystal Au nanowires. Nano Lett..

[12] Kim F, Sohn K, Wu J, Huang J (2008). Chemical synthesis of gold nanowires in acidic solutions. J. Am. Chem. Soc..

[13] Radha B, Kulkarni GU (2011). A real time microscopy study of the growth of giant Au microplates. Cryst. Growth Des..

[14] Wu X, Kullock R, Krauss E, Hecht B (2015). Single-crystalline gold microplates grown on substrates by solution-phase synthesis. Cryst. Res. Technol..

[15] Huang J, Lin L, Sun D, Chen H, Yang D, Li Q (2015). Bio-inspired synthesis of metal nanomaterials and applications. Chem. Soc. Rev..

[16] Hu H, Zhou JY, Kong QS, Li CX (2015). Two-dimensional au nanocrystals: shape/size controlling synthesis, morphologies, and applications. Part. Part. Syst. Char..

[17] Cai Ping, Zhou Shu-Mei, Ma De-Kun, Liu Shen-Nan, Chen Wei, Huang Shao-Ming (2015). Fe_2_O_3_-modified porous BiVO_4_ nanoplates with enhanced photocatalytic activity. Nano-Micro Lett..

[18] Li N, Zhao PX, Astruc D (2014). Anisotropic gold nanoparticles: synthesis, properties, applications, and toxicity. Angew. Chem. Int. Ed..

[19] Huang JS, Callegari V, Geisler P, Bruning C, Kern J (2010). Atomically flat single-crystalline gold nanostructures for plasmonic nanocircuitry. Nat. Commun..

[20] Deckert-Gaudig T, Deckert V (2009). Ultraflat transparent gold nanoplates: ideal substrates for tip-enhanced Raman scattering experiments. Small.

[21] Niu J, Wang D, Qin H, Xiong X, Tan P (2014). Novel polymer-free iridescent lamellar hydrogel for two-dimensional confined growth of ultrathin gold membranes. Nat. Commun..

[22] Chu HC, Kuo CH, Huang MH (2006). Thermal aqueous solution approach for the synthesis of triangular and hexagonal gold nanoplates with three different size ranges. Inorg. Chem..

[23] Wiley B, Sun Y, Mayers B, Xia Y (2005). Shape-controlled synthesis of metal nanostructures: the case of silver. Chemistry.

[24] Xiong Y, Washio I, Chen J, Cai H, Li ZY, Xia Y (2006). Poly(vinyl pyrrolidone): a dual functional reductant and stabilizer for the facile synthesis of noble metal nanoplates in aqueous solutions. Langmuir.

[25] Wang C, Kan C, Zhu J, Zeng X, Wang X, Li H, Shi D (2010). Synthesis of high-yield gold nanoplates: fast growth assistant with binary surfactants. J. Nanomater..

[26] Zhu W, Wu YY, Wang CY, Zhang M, Dong GX (2013). Fabrication of large-area 3-d ordered silver-coated colloidal crystals and macroporous silver films using polystyrene templates. Nano-Micro Lett..

[27] Liu B, Xie J, Lee JY, Ting YP, Chen JP (2005). Optimization of high-yield biological synthesis of single-crystalline gold nanoplates. J. Phys. Chem. B.

[28] Chen L, Ji F, Xu Y, He L, Mi Y, Bao F, Sun B, Zhang X, Zhang Q (2014). High-yield seedless synthesis of triangular gold nanoplates through oxidative etching. Nano Lett..

[29] Huang Y, Ferhan AR, Gao Y, Dandapat A, Kim DH (2014). High-yield synthesis of triangular gold nanoplates with improved shape uniformity, tunable edge length and thickness. Nanoscale.

[30] Millstone JE, Métraux GS, Mirkin CA (2006). Controlling the edge length of gold nanoprisms via a seed-mediated approach. Adv. Funct. Mater..

[31] Huang WL, Chen CH, Huang MH (2007). Investigation of the growth process of gold nanoplates formed by thermal aqueous solution approach and the synthesis of ultra-small gold nanoplates. J. Phys. Chem. C.

[32] Wang L, Chen X, Zhan J, Chai Y, Yang C, Xu L, Zhuang W, Jing B (2005). Synthesis of gold nano- and microplates in hexagonal liquid crystals. J. Phys. Chem. B.

[33] Hong S, Acapulco JAI, Jang H-J, Kulkarni AS, Park S (2014). Kinetically controlled growth of gold nanoplates and nanorods via a one-step seed-mediated method. Bull. Korean Chem. Soc..

[34] Kan C, Zhu X, Wang G (2006). Single-crystalline gold microplates: synthesis, characterization, and thermal stability. J. Phys. Chem. B.

[35] Millstone JE, Park S, Shuford KL, Qin L, Schatz GC, Mirkin CA (2005). Observation of a quadrupole plasmon mode for a colloidal solution of gold nanoprisms. J. Am. Chem. Soc..

[36] Siekkinen AR, McLellan JM, Chen J, Xia Y (2006). Rapid synthesis of small silver nanocubes by mediating polyol reduction with a trace amount of sodium sulfide or sodium hydrosulfide. Chem. Phys. Lett..

[37] Kumar-Krishnan S, Prokhorov E, Arias de Fuentes O, Ramírez M, Bogdanchikova N, Sanchez IC, Mota-Morales JD, Luna-Bárcenas G (2015). Temperature-induced au nanostructure synthesis in a nonaqueous deep-eutectic solvent for high performance electrocatalysis. J. Mater. Chem. A.

[38] Zeng J, Xia X, Rycenga M, Henneghan P, Li Q, Xia Y (2011). Successive deposition of silver on silver nanoplates: lateral versus vertical growth. Angew. Chem. Int. Ed. Engl..

[39] Heo J, Lee YW, Kim M, Yun WS, Han SW (2009). Nanoparticle assembly on nanoplates. Chem. Commun..

[40] Daniels JK, Chumanov G (2005). Nanoparticle-mirror sandwich substrates for surface-enhanced Raman scattering. J. Phys. Chem. B.

[41] Kim K, Yoon JK (2005). Raman scattering of 4-aminobenzenethiol sandwiched between Ag/Au nanoparticle and macroscopically smooth au substrate. J. Phys. Chem. B.

[42] Wu J, Xu Y, Xu P, Pan Z, Chen S, Shen Q, Zhan L, Zhang Y, Ni W (2015). Surface-enhanced Raman scattering from AgNP-graphene-AgNP sandwiched nanostructures. Nanoscale.

[43] Tang J, Ou FS, Kuo HP, Hu M, Stickle WF, Li Z, Williams RS (2009). Silver-coated si nanograss as highly sensitive surface-enhanced Raman spectroscopy substrates. Appl. Phy. A.

[44] Nie S (1997). Probing single molecules and single nanoparticles by surface-enhanced Raman scattering. Science.

[45] Jiang J, Bosnick K, Maillard M, Brus L (2003). Single molecule Raman spectroscopy at the junctions of large ag nanocrystals. J. Phys. Chem. B.

[46] Ji W, Xue X, Ruan W, Wang C, Ji N, Chen L, Li Z, Song W, Zhao B, Lombardi JR (2011). Scanned chemical enhancement of surface-enhanced Raman scattering using a charge-transfer complex. Chem. Commun..

[47] Cabalo J, Guicheteau JA, Christesen S (2013). Toward understanding the influence of intermolecular interactions and molecular orientation on the chemical enhancement of sers. J. Phys. Chem. A.

