# Supplementary material for: Rapid Seedless Synthesis of Gold Nanoplates with Microscaled Edge Length in a High Yield and Their Application in SERS
Source: Nanomicro Lett. 2016 May 11;8(4):328–35. doi: 10.1007/s40820-016-0092-6 (PMC6223685; doi:10.1007/s40820-016-0092-6)
Supplement: Supplementary file 1 — Supplementary material 1 (PDF 334 kb) [file 40820_2016_92_MOESM1_ESM.pdf]

Supporting Information for

Rapid Seedless Synthesis of Gold Nanoplates with Micro-scaled Edge Length in a High Yield and Their Application in SERS

Sheng Chen[1, 2], Pengyu Xu[1, 2], Yue Li,[1, 2] , Junfei Xue[1, 2], Song Han[2], Weihui Ou[2], Yaping Ding[1], Weihai Ni[2, *]

[1]Department of Chemistry, College of Sciences, Shanghai University, Shanghai 200444, People's Republic of China

Sheng Chen, Pengyu Xu, Yue Li, Junfei Xue, Song Han, Weihui Ou, Weihai Ni

[2]Division of i-Lab & Key Laboratory for Nano-Bio Interface Research, Suzhou Institute of Nano-Tech & Nano-Bionics, Chinese Academy of Sciences, Suzhou, Jiangsu 215123, People's Republic of China

*Corresponding author. E-mail: whni2012@sinano.ac.cn

Fig. S1 Statistical histogram of the edge length of triangular and hexagonal gold nanoplates

Fig. S2 a Extinction spectra of Au products grown at different temperature. The molar ratio of Br^-/Au is fixed at 6. **b** Those at different molar ratios of Br^-/Au. The reaction temperature is fixed at 85 °C

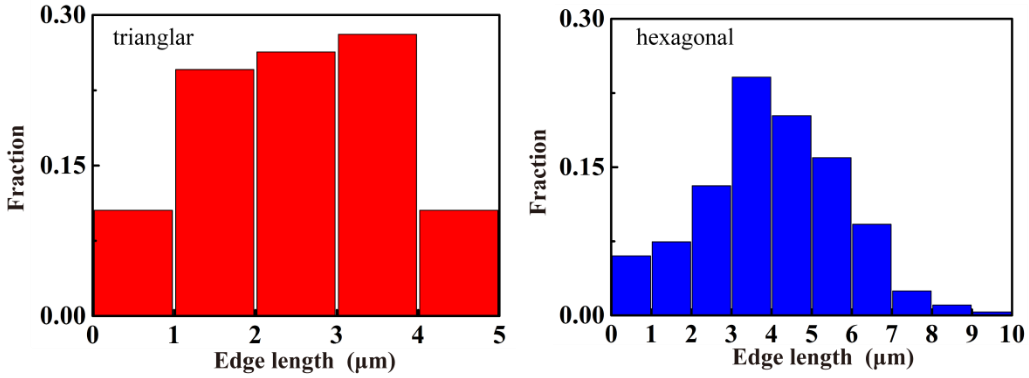
trianglar
hexagonal
Fraction
0.30
0.15
0.00
Edge length (μm)

Fig. S1

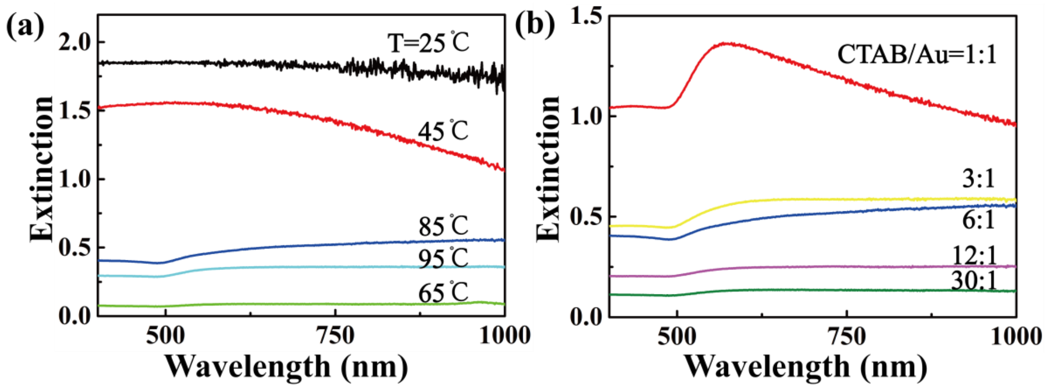
(a)
(b)
T=25℃
45℃
85℃
95℃
65℃
CTAB/Au=1:1
3:1
6:1
12:1
30:1
Extinction
Wavelength (nm)

Fig. S2
